# Asiatic Cholera, an Extract from a Paper Read before the Chicago Medical Society

**Published:** 1852-12

**Authors:** E. McArthur


					﻿ARTICLE II.—Asiatic Chalera, an extract from a paper read to the Chicago
Medical Society, by E. McArthur, M. D.—(Continued from page 304.)
Having formerly selected 34 cases from more than twice that
number of patients sick with Asiatic Cholera, and under my treat-
ment in the year 1849, and reported them somewhat in detail in
a paper read before the Chicago Medical Society two years since,
I propose now to continue the subject matter by reporting the fol-
lowing cases, selected with care, and with as much accuracy as
practicable:
I find by referring to my day and note-book, for the years
1850, 1851, and 1852—-the present year—that a large number of
patients sick with Asiatic Cholera have been under my treatment.
In the following tables, carefully prepared with reference to date
of attack, age, nativity, prominent symptoms, result as to recovery
or death, I have likewise showm the proportion of patients w’ho
had, or who had not been exposed to others sick with the same
disease previously to their attack. Of course this has been a deli-
cate as well as difficult task, being necessarily dependant in a good
degree upon the relations given by the patients themselves, their
friends and acquaintances, and what personal knowledge I might
possess in connection therewith. Those who have been engaged
in the same field of professional labor in this city with myself for
the past four years, or in other cities and localities where the
Cholera has extensively prevailed, will, I trust, appreciate the
difficulties in getting up such a report, and at the same time give
me credit for candor in making it.
I have also prepared tabular views of the number of families in
which these patients were sick, the number of subsequent attacks,
and whatever else might be deemed interesting in a practical ex-
amination of the subject, either to the Medical Profession, or to
the public at large.
It will be seen that we have selected forty-two cases under treat-
ment in the year 1850, eleven only in 1851, and fourteen the
present season. It may be well to premise that we saw and pre-
scribed for more patients sick with Cholera in each year than the
number reported. Also, that in 1851 my health was very poor,
and consequently my practice was more limited than before or
since that time.
K-'TIVITV. SYMPTOMS. J* 3„
i	44	k	-----------
.§	5	■§	&	&	• ■?
5	3	* s 2 a	ri u	* • -	i s ^	■
- o “	; | - a « { a j £ * J 17	h « |
----------- ---------------I 5	- J - i = j z 4 Ti ^ ;
1850.	--"	----
2	™y \5 ;5 da>' 1 da? 6~	9	0 0 0 0 o 0 .,	0 0
3	“	16 ri “I Rd*yS-20	0 o 0 u 0	o 0 J	o
.	io o | b *• <JU	0	n <i 0 0 n *	n i
4	“ 22 2 <• 2 “ 31 0	0 a 0 * a a ’
s “ » . I S 2 « a	o’ o ? ? 0 o 0 ’	’ 0 S’
9	‘; 29 7 “ a *‘ 23 0	9 9 n 0 '	9 9 0
I “ a 2 : t:
SilKKS S ;	: ’>»	•*
a “	u*«‘ 82 2.	0	: s’	- s"
1? :; ?■?;: «•; “ ’ „	SSSS s .; ;
“ «	IS - i - «	s	0 S	S S ’	' o ’ »
19	7 7 U 7 4, -LI A	0 0 U U I J u )
20	“	!2 1 “	1 “	30 0	0 n o 0 0 ° J n 3	° 9
S I 7 1 “ n	. o So o o s 0 0 J 999
23 « io t g ! 7 ?' °	a	0 0 0 0 0 o 0	0 ,j
I :.	-	-vir r	°o	Inr	*?77'^
1	::	i	& T*s	o us;	J°o j00
28	-	% ] 7	“ * 9	0 0 0 0 0	0 0 0 S °
29	g	no	7	,,	*	-“2	9	0	0	0	)	1	o
30	<. o? ?	5 davS 36 0	0 0	0 0 o	'	0	0
31	« at U *? n	oooooooo
32	«' i	■> “	>	«	o]	2	0 0	0	0	0	o 0	•	j
33	g	t ■<	7	<■	a!	°	0	9 0	0	0	0	0	0	)
34	<•	31	9 u	J	g	a?	0	9 0	0	0	0	oj	0	}
35	-en L. .:	-	0	9 0 0 0 0 0 0	0	0 )
36	•' 14 t « 1 g IS 0	9 9 0	0	0	0 )
37	«	23	1 «	•	))	m o	n 9	■»	9	9	0	0	0)
38	»	24	[ <•	7	i(	39	0 o	0	0	0	0	0	0
39	g	‘ a	1	4	0	0 °	o	0	0	0	0	0	9	0
40	«	2 » 2	‘	5	0	0 0 0 0 0	0	0	1	0
41	g	7 u 2	5	9	0 0 0 0 0	0	0	0 0
tt0e; 7	1,» Y 7 °	0 0 0 0 0	0	0 )	0
1851^ 6 h 4 hs- 3*	0	0 0 0 J 0	0	0	0)
2	AuJ X5 tdayS td??8 -a °	0 0 0 0 0 0.) Oo
t hrF.°	sssss •
I:: 5	S	•!::• .	?
s 7 s ’ »	„?«■’» 3	3 >“ ’•
9	18 2 “ 1	/	9 0 0 ) 0	oO 1 o
10	46pt 8 2 •< > g lo	0 9 9 9 9 9	0 0 0 )
11	• 21 4 “	•> S	0	O 0 o 0	o 0	0 0
1852	0 0 0)0	o )	.0 )
oJu‘yl 9	7	•	<’	S:	0 ■	oooooooo'o	o
3	g il b ‘ l : S 9 0	9 9	0	0 )	,
t	“	161	G	L.	o	S	s	°o 9 S	Sts	9
6	‘‘	19 1	t	f	t	?	S	0	0	0 0- 0	0).)	o °
7	g	20 i	g	4	g	a9	S	0	0	0 0 0	0 0)	£
9 Au-s 25 5 :■ ;:: 39 0	° 0 s S S S s s0 ?
liT Ug }g g S	99949 SJ.) S '0
11	13 3 “2 “ ■»»I I I ■»»? o |o | 8 :o ; $
* .Sick 4 days before I saw f 14th Aug., gave birth to a daughter—mother and phiiri ra J
t Father and daughter taken same day.	id done weU-
In order to make the above table easily understood and fully
practical, in elucidating the peculiar elements and circumstances
connected with the development of the disease here, so far as these
cases may go to show, I have added the following sub-tables, which
are principally collected from the foregoing :
No. 1—Time Sick.
No. sick less than one day, ------	3
“ sick one day, -------13
sick two days, -	--	--	--20
“	sick three to four days inclusive,	-	-	-	-	13
“	sick five to ten days inclusive,	-	-	-	-	15
“ sick over ten days, ------	3
Total,.................................67
No. 2—Age of Patients.
No. under ten years,	-	-	-	-	-	-12
“ ten, and less than twenty years,	4
“	twenty, and less than thirty years,	-	-	-	-	11
“	thirty, and less than forty years,	-	-	-	-	25
“ forty, and less than fifty years,	8
“ fifty, and less than sixty years,	3
“ sixty years, and upwards, -----	3
“ »age unknown,	-	-----	1
Total,.................................67
No. 3—Nativity.
United States,	-	-	35 Norway,	3
England,	-	-	-	3	Holland,	-	-	-	2
Ireland,	-	-	-	13	Wales,	-	-	-	1
Scotland,	-	-	-	2	Unknown,	-	-	-	1
Germany, -	-	-	9	—
Total, -	-	-	.-	-	-	67
No. 4—Symptoms.
No. Collapsed,	-	-	-	-	-	47
“ who vomited, -----	65
“	« Purged,	*	-	-	-	-	67
“	“	had “ Rice Water,”	_	-	-	65
“	“ Cramped, -	-	-	-	-	57
“	“	had Consecutive Fever,	-	-	-	13
No. 5—Exposure.
No. of Patients exposed, -	-	-	-	24
££	“	“ not exposed,	«■	-	-	42
“	“	“ unknown, -	-	-	_	1
Total,	-----	67
No. 6—Result to Patient.
No. who recovered,	-	-	-	-	-	30
No. who died,	-	-	-	-	-	-	37
Total,	-	-	-	-	-	-	67
No. 7—Sex.
No. Males,	-	-	-	-	-	-	30
No. Females,	-	-	-	-	-	-	37
Total,	-	-	-	-	_	.	67
/
No. 8—Families, &c.
No. of Families in which the sixty-seven cases occurred, -	53
££ of Families in which there was 2d attack, -	-	15
a	n	ii	ci	ci	“3d	“	-	-	5
“	“	“	“	“	“ 4th	“	-	-	2
££	££	££ where no subsequent attack,	-	-	48
No. 9—Relative Mortality of the Sexes.
No. of Males who lived,	14 No. of Males who died,	16
££ 11 Females who lived, 16	££ ££ Females who died, 21
30	37
But cases have been selected with reference to a knowledge
either personal or relative, in regard to their history.
Whatever views the Profession may have entertained in regard
to the contagious or non-contagious nature of Asiatic Cholera pre-
viously to its advent in this country in 1849, and its subsequent
continuation with short intervals only, up to the present time, it
would seem that enough has been seen of its progress and charac-
ter to justify the conviction in the mind of the candid inquirer,
that it should be classed with, and identified as strictly an epidem-
ic disease.
We may look at the subject in a negative light first, and it will
be difficult to point out a single distinctive feature in its onset and
progress, characterising contagious disease. In all the acknowl-
edged contagious diseases, or nearly so, there is a sthenic condi-
tion of the vital forces: the blood circulates with increased mo-
mentum ; the nervous energy is excited; sensibility is greatly in-
creased ; and all the elements and powers of excitation are aug-
mented. To those who have seen the onset and progress of Chol-
era, it will be unnecessary to say that not one of these circumstan-
ces characterise this disease.
On the other hand, the force of the pulsers diminished, and in
severe cases pulsation in the extremities entirely ceases; the ner-
vous energies are prostrated, and all the vital powers gradually
or rapidly waste away in proportion to its progress. If there are
symptoms pathognomonic .of any particular disease, those above
enumerated may be fairly considered as such in regard to contagious
diseases in contradistinction to this.
Most diseases of a contagious character and origin, seldom if
ever attack the same person the second time; but on the other
hand, having had the Cholera once, is no more immunity against a
second attack, than once having the Intermittent Fever renders the
individual incapable of being affected by it again. Indeed, there
is much similarity in the way of commencement in these last two
diseases, in the principles connected with their attack upon persons
and communities. A simple change in the atmosphere is all that
is necessary either to cause them to spread with fearful rapidity,
or check their progress, and even to restore perfect health to soci-
ety. There appears to be quite as much evidence of the one being
contagious as there is of the other being so: the obscurity in re-
lation to the predisposing cause having never been fully and prac-
tically obliterated, so that our senses could be cognisant of a mate-
rial and palpable cause for either Intermittent Fever or Asiatic
Cholera. And yet by common consent mankind have acquiesced
in the opinion that a latent principle was at times concealed in the
atmosphere, which caused such wide-spread affliction and suffering
in human society by the one or the other of these forms of dis-
ease. In what respect a little appreciable change in the temperature
humidity, or other inappreciable alteration of the air, should affect
the liability of persons and communities to imbibe contagious dis-
cases, and so suddenly, is an inquiry worthy of solution by those
writers who contend that Asiatic Cholera is contagious.
But we will now turn our attention to the consideration of Chol-
era as it has been developed in this city, without any further pre-
lude.
In 1849, the first and second cases of this disease which fell un-
der our own personal observation, had not been exposed in the least
to any person having it, or having had it. This we learned by a
rigid and thorough inquiry made at the time. And it will be seen,
by reference to a former paper upon this subject, that in twenty-
five families where this disease was found, sixteen did not have a
subsequent attack. In the first case, as well as in a majority of
the forty-two cases reported for 1850, there had been no exposure
to persons sick with the disease. And by reference to the princi-
pal table in this communication, it will be seen that, in the eleven
cases reported for 1851, there were but two who had previously
been exposed, and there were but two subsequent attacks in the
families where these eleven were sick. In the fourteen cases re-
ported as having been sick with the disease the present year, seven
only had been exposed ; and in the families where eight out of the
original number had been living, there has been no subsequent at-
tack.
There certainly is no apparent resemblance in this statement of
facts and view of the subject, to the effects and results of any con-
tagious disease with which we have been acquainted. There are
other views and illustrations in connection with the prevalence of
this disease in this city and section of country which cannot be
given in a tabular form, nor in the report of cases, even should
they be greatly multiplied.
For these four years now last past, there has been a striking
similarity in the circumstances attending the commencement of
the disease in each year. For some days previous to its break-
ing out there has been an appreciable change of the atmosphere,
which is easily felt and understood by those who have previously
felt its influence and seen the consequences resulting therefrom.—
Simultaneously with this atmospheric change we have had an al-
most universal tendency to derangement of the stomach and bow-
els. The appetite has been very suddenly affected in many cases:
in more, however, there has been flatulency of the bowels and diar-
rhoea. With many this diarrhoea has been of a bilious character,
but very soon passing into that of a serous kind. In other instan-
ces the diarrhoea has been of a serous character from the, com-
mencement. This peculiar condition of the system has been felt
for some time by a large portion of community, varying from one
day to several, when all at once individuals have been attacked with
Cholera in every ward in the city simultaneously, and in some in-
stances in different parts of each ward. This general development
of the disease in our midst has been at times after a few’ scattering
cases. In other instances it has broken forth promiscuously through
the city, where there had been scarcely a patient sick with Cholera
for several weeks. On the night of the 13th of October of the
present year, there w’ero sudden attacks of Cholera in every ward
of the city, attended with quite a number of deaths, and yet there
had been very little of the disease in the city for a number of w’eeks
previously, and most of what had been was confined exclusively to
the foreign population. And but few if any cases occurred for
weeks afterwards. And generally when this sudden breaking forth
of the disease lias been so fearfully manifested in our midst, not
one in five—perhaps not one in ten—have been exposed in any way
to those affected by it. It has furthermore been observed by most
practitioners conversant with this disease, that oftentimes one or
more persons have had it, and very seriously too—in many cases
death being the result to the patient—in different families here and
there ; and although the balance of the families and numerous
friends and visiters have been equally exposed to the sick, there has
been no subsequent attacks which can be traced to this exposure.
Indeed, if we can form any estimate of the liability of persons to
an attack from this disease, by the experience of the four years pas 1
in which it has prevailed here, we can say with confidence that any
person is less likely to be taken with it, however much exposed, if
that person is calm, quiet, regular in general habits, temperate, &c.,
than another person who is fearful, excited, irregular in habits, in-
temperate, &c., even though he may not have been near any per-
son having the disease, or in any other way have been exposed.
When Cholera prevails, a sudden change in the atmosphere for
the better causes a corresponding improvement in the public health.
It is really atmospheric influence, and not exposure, which causes
the breaking forth of this fearful scourge. And it is appreciable
improvement, too, in the atmosphere which causes its subsidence,
and not want of exposure. In this city Cholera has prevailed more
extensively in low sections and neighborhoods, and especially if
the inhabitants have used exclusively water from the wells, which
are nothing more or less than reservoirs of surface water. There has
been an appreciable difference in this respect in regard to locality.
Those families using hydrant water (brought in pipes from Lake
Michigan,) have suffered less—all other circumstances being equal
—than those which have used well water. In this respect like-
wise there is a striking similarity in the causes which govern the
development of the two diseases—Asiatic Cholera and Intermittent
Fever. Those Physicians who practice in malarious districts well
know the difference in the prevalence of Intermittents in low or
elevated sections, and in the use of well water from low, marshy
places, or good spring water.
The inhabitants living in this city who are temperate and
prudent in their habits, regular in their calling and business, and
at the same time avoid excitement, alarm, &c., have been compar-
atively exempt from attacks of Cholera; while foreigners who have
recently come among us, the intemperate, the fearful, the poor fed
and excited, have been exceedingly prone to it. Physicians who
have had the most experience in practice with foreigners and pau-
pers in the city, are almost universally inclined to the belief that
with these classes of patients Cholera is rather the result of in-
temperance, "want, irregularity, fear, &c., operating as an exciting
cause, than to any other known cause. And most of these prac-
titioners are fully persuaded that an epidemic influence operates as
a predisposing cause, rather than that the disease depends in any
respect upon a contagious influence.
				

